# Validity of a Local Positioning System during Outdoor and Indoor Conditions for Team Sports

**DOI:** 10.3390/s20205733

**Published:** 2020-10-09

**Authors:** Prisca S. Alt, Christian Baumgart, Olaf Ueberschär, Jürgen Freiwald, Matthias W. Hoppe

**Affiliations:** 1 Department of Orthopaedic and Traumatic Surgery, Clinic Braunschweig, 38118 Braunschweig, Germany; 2 Department of Movement and Training Science, University of Wuppertal, 42119 Wuppertal, Germany; baumgart@uni-wuppertal.de (C.B.); freiwald@uni-wuppertal.de (J.F.); 3 Department of Engineering and Industrial Design, Magdeburg-Stendal University of Applied Sciences, 39114 Magdeburg, Germany; ueberschaer@iat.uni-leipzig.de; 4 Department of Biomechanics, Institute for Applied Training Science, 04109 Leipzig, Germany; 5 Institute of Movement and Training Science I, University of Leipzig, 04109 Leipzig, Germany; matthias.hoppe@uni-leipzig.de

**Keywords:** acceleration, accuracy, football, handball, player tracking technology, soccer

## Abstract

This study aimed to compare the validity of a local positioning system (LPS) during outdoor and indoor conditions for team sports. The impact of different filtering techniques was also investigated. Five male team sport athletes (age: 27 ± 2 years; maximum oxygen uptake: 48.4 ± 5.1 mL/min/kg) performed 10 trials on a team sport-specific circuit on an artificial turf and in a sports hall. During the circuit, athletes wore two devices of a recent 20-Hz LPS. From the reported raw and differently filtered velocity data, distances covered during different walking, jogging, and sprinting sections within the circuit were computed for which the circuit was equipped with double-light timing gates as criterion measures. The validity was determined by comparing the known and measured distances via the relative typical error of estimate (TEE). The LPS validity for measuring distances covered was good to moderate during both environments (TEE: 0.9–7.1%), whereby the outdoor validity (TEE: 0.9–6.4%) was superior than indoor validity (TEE: 1.2–7.1%). During both environments, validity outcomes of an unknown manufacturer filter were superior (TEE: 0.9–6.2%) compared to those of a standard Butterworth filter (TEE: 0.9–6.4%) and to unprocessed raw data (TEE: 1.0–7.1%). Our findings show that the evaluated LPS can be considered as a good to moderately valid tracking technology to assess running-based movement patterns in team sports during outdoor and indoor conditions. However, outdoor was superior to indoor validity, and also impacted by the applied filtering technique. Our outcomes should be considered for practical purposes like match and training analyses in team sport environments.

## 1. Introduction

Presently, player’s physical tracking is indispensable in both outdoor and indoor team sports [1,2,3]. By the continuous monitoring of match and training activities, the training load [4], regeneration process [5], injury prevention [6,7,8], and on-field rehabilitation can be optimized. During the past decade, the technological development occurred fast, so that several player-tracking technologies are available today. Whilst initially camera-based technology was used, global (GPS) and local (LPS) positioning systems have become standard technologies [2,9]. However, comprehensive evaluations of both technologies are essential to allow a valid interpretation of the data acquired [9,10,11].

In this context, many studies summarized into one review [12] have investigated the validity and reliability of GPS for measuring distances covered, speed, and acceleration [7,13,14,15,16,17,18,19,20,21,22,23,24]. These studies show that GPS allows an accurate determination of total distances covered; however, the accuracy is compromised during accelerations, change of directions, and sprints [2,9]. A further limitation of GPS is that the technology is limited to outdoor sports due to its satellite-based measurement principle. Instead, LPS grounds on a local network of antennas and transponders, and therefore can be used in both outdoor and indoor conditions [25]. To date, compared to GPS, there are clearly fewer studies regarding the validity and reliability of LPS for team sports [9,25,26,27,28,29,30,31]; although, the first review has recently been published in that regard [32]. In line with GPS, those studies demonstrate that LPS enables to accurately assess average speed data [21,32], but limitations are present in peak acceleration/deceleration [29,32], instantaneous speed [11,30,32], and change of direction [27,32] measures. However, it is worth mentioning that LPS accuracy during a team sport-specific circuit, involving for soccer, handball, etc. typical movement patterns, is superior to that of GPS [9].

Due to its local measurement principle, LPS is presently used in several indoor sports on a daily basis. For example, in the German top national level Handball-Bundesliga, European Handball Federation, NBA G League, and German Ice Hockey League. In the present Covid-19 crisis, it is also applied to keep distance and to trace infection chains in the industrial sector [33]. Thus, more studies to evaluate the LPS technology are required; especially, due to its increased use during indoor conditions. To the best of our knowledge, there is only one study that compares the validity of a LPS during outdoor and indoor conditions. That study reported a higher distance error during indoor (2.0–2.4%) than outdoor (1.3–1.4%) conditions [25]. Unfortunately, the data were only collected under ideal conditions in the centre of the antenna sourcing field, where the position detection functions optimally [30]. Additionally, and not in line with a plethora of previous GPS and LPS validation studies [2,7,9,11,21,25,26,27,28,29,30,34], no specific results regarding the impact of different accelerations, changes of direction, and speeds were shown. Thus, a validation study to address these points is required. Since it has also been shown that LPS-based speed data are impacted by more noise during a team sport-specific circuit than GPS-based data [9], it is also worth to examine the effect of different filtering techniques on the LPS accuracy, which has not been conducted so far. In this context, a rational approach is to compare the impact of standard filtering techniques with those developed by the manufactures, because the latter are used by the end consumer in practice [35].

This study aimed to compare the validity of a LPS during outdoor and indoor conditions for team sports. The impact of different filtering techniques was also investigated. Based on previous studies, it was hypothesized that LPS validity is superior during outdoor compared to indoor conditions [25] and that the filtering technique thereby has an impact [9].

## 2. Materials and Methods

### 2.1. Subjects

In our study, five male team sport athletes (age: 27 ± 2 years; height: 1.77 ± 0.04 m; mass: 79.2 ± 2.2 kg; fat: 14.7 ± 3.7%; maximum oxygen uptake: 48.4 ± 5.1 mL/min/kg; 3–4 training sessions per week) took part. All athletes participated in team sports (American football: n = 1, basketball: n = 1, and soccer: n = 3) and competed on an adult amateur playing level. The athletes were informed of the purposes and potential risks of the study procedures and signed a written informed consent to participate. All procedures were pre-approved and accepted by the Ethics Committee of the local university (MS/JE 29.11.11) and were conducted in accordance with the Declaration of Helsinki.

### 2.2. Design

**Figure 1:**
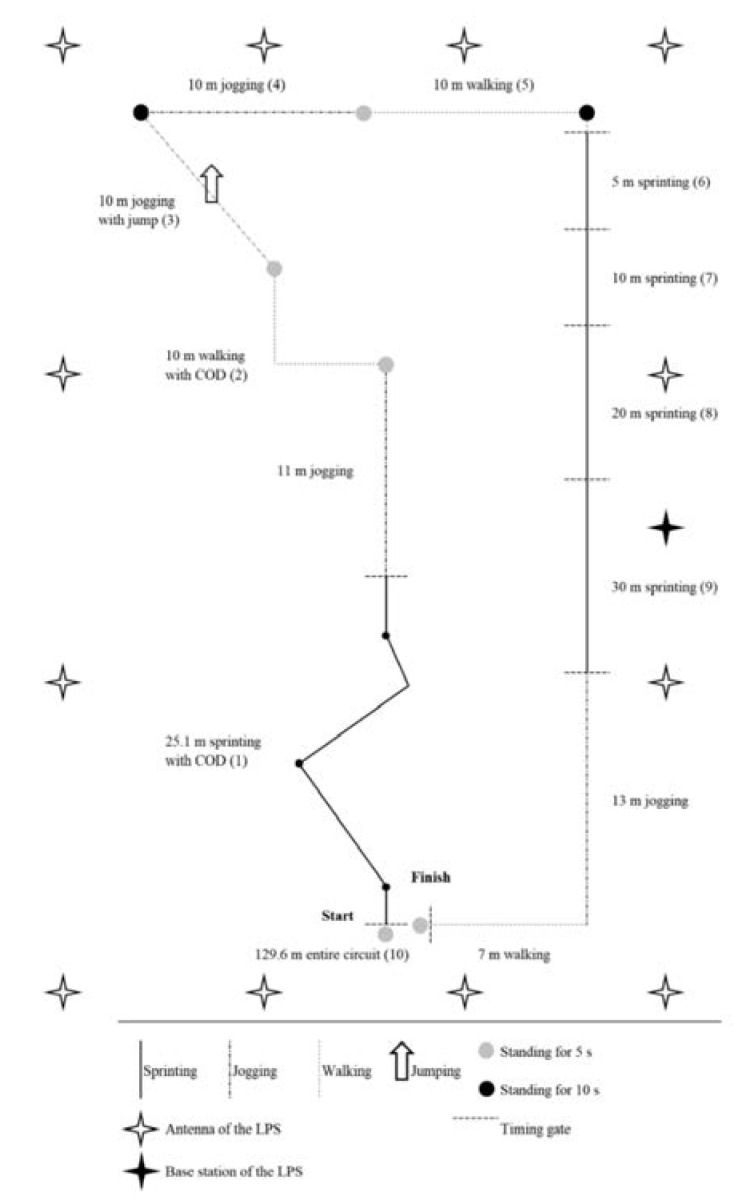
The design of the circuit and the setup of the local positioning system. Note: LPS = Local positioning system; COD = Change of direction. The particular sections that were used for determining the distances covered were numbered from 1 to 10 (in brackets) to allow a better assignment of the results provided in the tables and figures.

All athletes performed 10 valid trials of a team sport-specific circuit during outdoor and indoor conditions, which were separated by one week. A trial was considered as valid, if the athletes had completed the circuit without disruptions and if there was no self-inflicted measurement error. The measures took place on a dry artificial turf soccer field and a standard sports hall used for official handball competitions. The circuit included different walking, jogging, sprinting, and standing sections over an entire distance of 129.6 m, as described previously [9]. All sprinting sections were equipped with double-light timing gates (Werthner Sport Consulting, TDS, Linz, Austria) [35] as criterion measures. Thus, the criterion validity was determined here. During the circuit, the athletes wore artificial turf and indoor shoes during outdoor and indoor conditions, respectively, and two devices of one LPS between the scapulae in a custom-made sport shirt [36]. To investigate the validity, the known distances of the circuit were compared to the measured distances of the LPS. The capacity of the athletes to repeat the circuit during outdoor and indoor conditions was estimated by the timing gate measures, meaning that the test-retest reliability was quantified. Additionally, physiological responses were assessed by heart rate and ratings of perceived exertion measures. Figure 1 shows the design of the circuit and setup of the LPS.

### 2.3. Local Positioning System and Data Processing

A recent 20-Hz LPS (KINEXON Precision Technologies, KINEXON ONE, version 1.0, Munich, Germany) [36] was used. The system consists of one base station and 12 antennas each positioned at four meters above the ground around the circuit (Figure 1). One technician from the manufacturer performed all installations and calibrations. The measurement principle of the system is as follows: The sensors worn by the athletes send continual signals via radio waves to the antennas, which forward them via a wide local area network to the base station. The latter transforms the received data into the x- and y-positions of the athletes [9]. The position data were exported from the proprietary software (KINEXON Precision Technologies, Kinexon Web Application, version 3.2.6, Munich, Germany [36]) to custom-made spreadsheets involving macro-based calculations (Microsoft, Excel 2016, Redmond, WA, USA) for further data processing according to previous studies [9]: First, by numerical differentiation over time, the instantaneous speed data of the athletes were computed. Then, the speed data were passed through a second-order Butterworth low-pass filter with a 1-Hz cut-off frequency and two passes to minimize noise. From the filtered speed data and their integration over time, the distances covered during each section of interest within the circuit were computed. To detect the start and end of the walking, jogging, and sprinting sections, a speed threshold of 0.2 m/s was used. The end of each sprinting section was obtained from the timing gate information.

Since all athletes wore two sensors and performed 10 valid trials of the circuit during outdoor and indoor conditions, a total of 100 data files per condition were acquired. All data files were visually checked for measurement errors caused by poor signal strength or timing gate errors by two investigators, who did this independently of each other. Additionally, outliers were objectively defined as those values that were greater than the mean ± the two-fold pooled standard deviation [18]. A total of 20 and 11 data files were classified as outliers during outdoor and indoor conditions, respectively, and were removed from the statistical analyses. To investigate the impact of different filtering techniques, all finally included data files were also passed through an unknown filter of the LPS manufacturer, which is included in the company’s proprietary software and used by the end consumers in practice. The unprocessed raw data were also considered.

### 2.4. Statistical Analyses

At first, descriptive data were reported as means and standard deviations. Secondly, the relative typical error of estimate (TEE) in percent was calculated. The TEE was rated as good (0 to <5%), moderate (5 to <10%), or poor (≥10%) [7]. Differences in means were studied by Magnitude-Based Inferences. Therefore, the mean differences and their 90% confidence intervals were analyzed in relation to the smallest worthwhile differences (SWD), as described before [10]. The SWD was defined as the pooled standard deviation multiplied by 0.2 [10]. Then, the probabilities for the mean differences “truly” being higher than the associated SWDs were determined and qualitatively described using the following scale: <1%, most unlikely; 1 to <5%, very unlikely; 5 to <25%, unlikely; 25 to <75%, possibly; 75 to <95%, likely; 95 to <99%, very likely, and ≥99%, most likely. If the likelihoods for being both higher and lower were ≥5%, the differences were described as unclear. To also clarify the magnitudes, effect sizes according to Cohen’s d were calculated. The effect sizes were interpreted accordingly: 0.0 to <0.2, trivial; 0.2 to <0.6, small; 0.6 to <1.2, medium; 1.2 to <2.0, large; 2.0 to <4.0, very large; and ≥4.0, extremely large. For all statistical analyses, pre-built spreadsheets (Microsoft, Excel 2016, Redmond, WA, USA) were used.

## 3. Results

### 3.1. Reliability

Table 1 shows all descriptive data for the timing gate and physiological measures during outdoor and indoor conditions. Additionally, the results of the Magnitude-Based Inferences and corresponding effect sizes are reported. There were trivial to moderate differences between both conditions (unclear to very likely probabilities).

### 3.2. Descriptive Data

Table 2 summarizes all descriptive data concerning the measured distances by the LPS during outdoor and indoor conditions. The peak speed and acceleration data during the two sprinting sections are also shown. All data are presented with respect to the two different filtering techniques and as raw data.

### 3.3. Validity

Table 3 displays the calculated relative TEEs according to the outdoor and indoor conditions, and also to the different filtering techniques. During both conditions, the TEEs ranged from good to moderate (TEE: 0.9–7.1%). The overall TEEs for the filtering techniques and raw data were all good (TEE: 1.8–2.9%). However, the manufacturer filter showed the smallest overall TEEs during both conditions.

**Figure 2:**
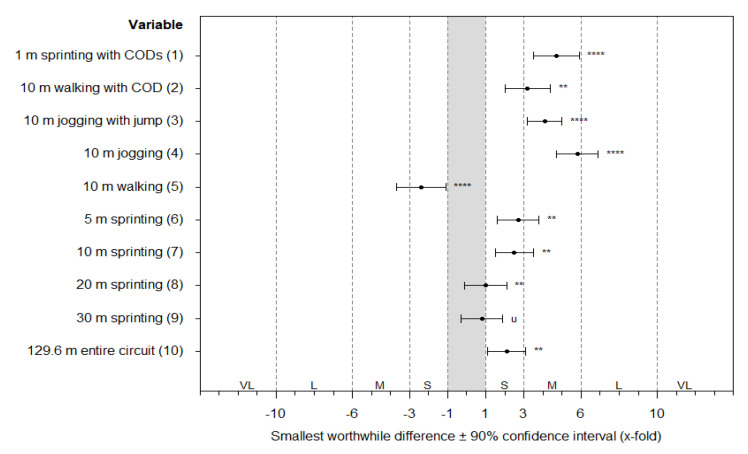
Effects of indoor vs. outdoor condition on distances covered measured by the local positioning system. For that comparison, the data of the manufacturer filter were used due to its highest validity outcomes (Table 3). Note: Each effect is shown as factor of the smallest worthwhile difference (SWD). The corresponding effect size thresholds for small (S; 1-fold), moderate (M; 3-fold), large (L; 6-fold), and very large effects (VL; 10-fold) are also shown. The asterisks *, **, ***, and **** indicate the probabilities that the effect is possibly (>75%), likely (>90%), very likely (>95.5%), and most likely (=100%) higher or lower than the SWD. The letter u indicates an unclear effect with probabilities of >5% that the effect is both higher and lower than the SWD. The numbers in the brackets present the section of measurement within the circuit (Figure 1).

Figure 2 shows the results of the Magnitude-Based Inferences concerning the differences in the distances covered between the outdoor and indoor conditions. For that comparison, the data of the manufacturer filter were used due to its highest validity outcomes (Table 3). There were trivial to moderate differences between both conditions (unclear to most likely probabilities).

## 4. Discussion

This study aimed to compare the validity of a LPS during outdoor and indoor conditions for team sports. The impact of different filtering techniques, namely that of a standard Butterworth and unknown manufacturer filter included in the company’s proprietary software, was also investigated. Based on previous studies, it was hypothesized that LPS validity is superior during outdoor compared to indoor conditions [25] and that the filtering technique thereby has an impact. Our main findings were that the LPS validity for measuring distances covered in team sports was (i) good-to-moderate during both environments, whereby the outdoor was superior than indoor validity; and (ii) impacted by the filtering techniques, showing superior validity outcomes for the manufacturer filter compared to those for the standard Butterworth filter and to unprocessed raw data.

To allow comparisons of the LPS validity during outdoor and indoor conditions, a methodological prerequisite is that the athletes reproduce the movement patterns with the same effort [10,37]. Compared to a previous study [25], a methodological strength of our study was that we have determined the capacity of the athletes to reproduce the circuit and also their physiological responses by timing gates, heart rate, and perceived exertion measures. Our data show only trivial to moderate differences between both environments (Table 1). Potential explanations for these differences are beside technological and biological sources [25,26] also the different ground surfaces of both environments. Since it is known that a hall floor has more grip than an artificial turf [38], it is plausible that our athletes were slightly faster (up to small effect sizes) during indoor conditions (Table 1). However, together with the physiological responses, our reliability findings overall demonstrate that our athletes were able to reproduce the movement patterns during both environments with a comparable effort.

Our first major finding was that the LPS validity for measuring distances covered in team sports was good to moderate during both environments (TEE: 0.9–7.1%), while the outdoor validity (TEE: 0.9–6.4%) was superior than indoor validity (TEE: 1.2–7.1%) (Table 3). Our validity outcomes are supported by those of previous studies [2,9,11,21,25,26,27,28,29,30,34]. These studies evaluated the LPS validity predominantly during outdoor conditions for measuring different products as distances covered (TEE: 0.8–6.0%) [9,25,31] and further indicators with diverse statistical calculations allowing no direct comparison, as instantaneous speeds [27,29,30] and x-, y-positions [11,27,28,30]. Our data also reinforce an important issue that applies not only to LPS [11,21,26,29], but also to GPS [14,18,26,39]: The validity is compromised during accelerated runs over short distances [9,11,29], as shown most clearly by our validity indicators over the 5-m sprinting distance (TEE: 4.1–7.1%) (Table 3). When taken together, our and previous findings show that LPS can be considered as a good to moderate valid technology to assess running-based movement patterns in team sports.

A new outcome of our study was that the LPS validity was superior during the outdoor (TEE: 0.9–6.4%) than indoor condition (TEE: 1.2–7.1%) (Table 3). To the best of our knowledge, there is only one previous study that has also compared the LPS validity during both environments [25]. Although the research designs differ in terms of the evaluated technologies, movement patterns, data processing, and statistical analyses, the main finding in both studies is similar: The overall error is approximately one percentage point higher during the indoor (previous study: 2.0–2.4%; our study: 2.5–2.9%) than the outdoor (previous study: 1.3–1.4%; our study: 1.8–2.2%) condition. Taking the evidence together, the results show that the LPS validity is negatively impacted by the indoor environment. The overall lower indoor validity can be explained by signal interference mainly due to reflection, which may result from construction materials like metal objects in sports halls [9,25,30]. To clarify this assumption, more research is needed. Thereby, and for practical considerations, the numerous different structural conditions of sports halls have to be considered.

The second main outcome of our study was that the LPS validity for measuring distances covered in team sports was impacted by the filtering techniques. In fact, during both environments, the validity outcomes of the manufacturer filter were superior (TEE: 0.9–6.2%; overall: 1.8–2.5%) compared to those of the standard Butterworth filter (TEE: 0.9–6.4%; overall: 2.7–2.8%) or unprocessed raw data (TEE: 1.0–7.1%; overall: 2.2–2.9%) (Table 3). As known from GPS studies [35], the filtering technique is a crucial step during data processing. Indeed, different filtering techniques lead up to very large differences in the apparent number of acceleration events performed during soccer matches [40]. This outcome is in line with our found impacts on peak acceleration/deceleration data (Table 2). In this context, the unphysiologically high peak acceleration/deceleration data of the unprocessed raw data appear noteworthy (Table 2), which underlie the fundamental requirement to apply appropriate filtering techniques to the raw data before acceleration/deceleration data are computed [41]. With this in mind, there is still a surprising lack of research regarding the impact of filtering techniques on GPS and LPS validity. In a previous study, we showed that LPS was impacted by more noise than GPS (very large effect sizes), and also that the amount of noise was largest at the corners of the circuit close to the reference antennas [9]. Higher error rates of LPS data close to walls and corners have also been reported in further studies, and were related to undetected anchor nodes and multipath propagation [30,42]. Thus, filtering techniques may be of particular importance for the LPS validity during outdoor and indoor condition, and require more research clearly. The underlying explanations for the different impacts of the filtering techniques found on our LPS validity remain unclear. While we have applied a standard Butterworth filter from the field of biomechanics [43], the exact mathematical functioning and specifications of the used manufacturer filter are unfortunately unknown. The reason to investigate the impact anyway was that the filtering technique grounds on the best practice experience of the manufacturer and is included in the proprietary software used by the end consumers in daily practice. Thus, to progress the entire field of the player tracking technologies forward, we strongly encourage manufacturers to disclose their algorithms and filtering techniques [30,35,44]. In this context, it is also worth questioning, if raw data reported by proprietary software can been considered as “true” raw data, as conducted here, because it is likely that they have already been processed by further unknown filters working directly on the hardware level.

A potential limitation of our study is that we have used defined distances covered together with timing gates as a criterion measure. According to a previous validation study, there are three different validation levels: position, instant speed and acceleration, and from the latter aggregated data as distances covered [11]. Our study corresponds to the third level. To take also the validity of the position and underlying speed data into account, a camera-based reference system should have been used [11,30]. The main reason for our approach was that camera-based systems allow to assess speed data only within relatively small areas, and thus are unable to investigate movement patterns over longer distances at maximal speeds that are also part of team sports. Additionally, and due to the standard application of the LPS evaluated here, for example during each competitive match in the German Handball-Bundesliga, we aimed to evaluate the technology in external valid conditions that take the entire playing fields of team sports as handball into account, including the corners being challenging for position detection [9,27,30,42], which is presently not possible by a camera-based approach. However, to allow a better comparison of the findings derived by future validation studies, accepted methodological standards have to be developed, as recently summarized in first guidelines [45].

## 5. Conclusions

In conclusion, our study shows that the evaluated LPS can be considered as a good to moderately valid tracking technology to assess running-based movement patterns during outdoor and indoor conditions in team sports. However, it must also be concluded that the LPS validity was superior outdoor compared to indoor, and impacted by the applied filtering techniques, showing best validity outcomes for an unknown manufacturer filter. Our outcomes should be considered for practical purposes like match and training analyses in the team sport environment.

## Figures and Tables

**Table 1:** Timing gate and physiological data of the athletes during outdoor and indoor conditions.

Variables	Descriptive Data (Mean ± SD)		Effect Size	Magnitude-Based Inferences			
	Outdoor (n = 80)	Indoor (n = 89)	d	Descriptor	SWD	SWD ± 90% CI (x-Fold)	Descriptor
25.1 m sprinting with CODs (1) (s)	6.03 ± 0.27	5.95 ± 0.25	0.32	small	0.05	−1.3 ± 1.0	*
5 m sprinting (6) (s)	1.10 ± 0.05	1.10 ± 0.06	0.06	trivial	0.01	1.0 ± 1.1	u
10 m sprinting (7) (s)	1.85 ± 0.09	1.85 ± 0.09	0.07	trivial	0.02	0.5 ± 1.0	**
20 m sprinting (8) (s)	3.17 ± 0.15	3.14 ± 0.14	0.19	trivial	0.03	−0.1 ± 1.0	*
30 m sprinting (9) (s)	4.43 ± 0.20	4.38 ± 0.21	0.25	small	0.04	−0.4 ± 1.0	*
129.6 m entire circuit (10) (s)	97.45 ± 3.94	95.30 ± 3.32	0.59	small	0.73	−2.7 ± 0.8	***
HR (bpm)	159.6 ± 9.8	154.7 ± 10.2	0.42	small	2.4	−2.1 ± 1.9	u
RPE (1–20)	14.6 ± 1.0	13.4 ± 1.2	1.09	moderate	0.2	−5.5 ± 3.3	**

Note: SD = Standard deviation; n = number; d = Cohen’s d effect size; SWD = Smallest worthwhile difference; CI = Confidence interval, HR = Heart rate; RPE = Rating of perceived exertion. The asterisks *, **, ***, and **** indicate the probabilities that the effect is possibly (>75%), likely (>90%), very likely (>95.5%), and most likely (=100%) higher or lower than the SWD. The letter u indicates an unclear effect with probabilities of >5% that the effect is both higher and lower than the SWD. The numbers in the brackets present the section of measurement within the circuit (Figure 1).

**Table 2:** Descriptive data (mean ± SD) of the local positioning system for determining the distances covered during outdoor and indoor conditions. The impacts of the different filtering techniques, and the outcomes of the raw data, are also shown.

Variable	Outdoor (n = 80)			Indoor (n = 89)		
	Raw Data	BW 1 Hz	Manufacturer	Raw Data	BW 1 Hz	Manufacturer
25.1 m sprinting with CODs (1) (m)	24.7 ± 0.4	24.7 ± 0.4	22.7 ± 0.4	24.5 ± 0.5	24.5 ± 0.5	23.1 ± 0.4
10 m walking with CODs (2) (m)	10.5 ± 0.2	10.6 ± 0.2	10.1 ± 0.2	10.5 ± 0.3	10.5 ± 0.3	10.2 ± 0.2
10 m jogging with jump (3) (m)	10.4 ± 0.2	10.4 ± 0.2	10.2 ± 0.1	10.4 ± 0.2	10.5 ± 0.3	10.3 ± 0.2
10 m jogging (4) (m)	10.2 ± 0.2	10.6 ± 0.6	10.0 ± 0.1	10.3 ± 0.3	10.5 ± 0.3	10.2 ± 0.3
10 m walking (5) (m)	10.5 ± 0.3	10.9 ± 0.7	10.1 ± 0.1	10.1 ± 0.2	10.2 ± 0.2	10.0 ± 0.1
5 m sprinting (6) (m)	5.0 ± 0.2	5.0 ± 0.2	4.8 ± 0.2	5.0 ± 0.3	5.0 ± 0.3	4.9 ± 0.3
10 m sprinting (7) (m)	10.2 ± 0.3	10.2 ± 0.3	9.9 ± 0.3	10.2 ± 0.3	10.3 ± 0.4	10.0 ± 0.4
20 m sprinting (8) (m)	20.5 ± 0.5	20.5 ± 0.4	20.2 ± 0.4	20.4 ± 0.5	20.4 ± 0.4	20.2 ± 0.4
30 m sprinting (9) (m)	30.5 ± 0.3	30.6 ± 0.3	30.2 ± 0.3	30.4 ± 0.5	30.4 ± 0.4	30.2 ± 0.4
129.6 m entire circuit (10) (m)	139.0 ± 2.3	139.3 ± 2.1	130.8 ± 1.5	135.9 ± 3.0	136.6 ± 2.7	131.1 ± 2.4
Noise during standing (10) (m)	8.0 ± 1.4	7.0 ± 0.9	3.5 ± 0.45	6.5 ± 0.8	6.0 ± 0.7	3.7 ± 0.5
Peak speed (1) (m/s)	6.3 ± 0.7	5.7 ± 0.3	5.3 ± 0.20	5.8 ± 0.3	5.5 ± 0.2	5.2 ± 0.2
Peak acceleration (1) (m/s^2^)	33.6 ± 18.4	4.1 ± 0.5	4.5 ± 0.63	26.0 ± 14.7	4.1 ± 0.5	5.5 ± 0.8
Peak deceleration (1) (m/s^2^)	−24.8 ± 16.0	−3.2 ± 0.4	−4.7 ± 0.66	−18.6 ± 8.3	−3.3 ± 0.3	−5.4 ± 0.7
Peak speed (6–9) (m/s)	8.9 ± 0.7	8.2 ± 0.4	8.1 ± 0.41	8.6 ± 0.6	8.3 ± 0.4	8.2 ± 0.4
Peak acceleration (6–9) (m/s^2^)	46.1 ± 28.2	4.8 ± 0.4	5.4 ± 0.53	28.7 ± 23.4	4.9 ± 0.4	6.3 ± 0.9

Note: SD = Standard deviation; n = number; BW = Butterworth; COD = Change of direction. The numbers in the brackets present the section of measurement within the circuit (Figure 1).

**Table 3:** Relative typical error of estimates (mean ± SD) of the local positioning system for determining the distances covered during outdoor and indoor conditions. The impacts of the different filtering techniques, and the outcomes of the raw data, are also shown.

Variable	Outdoor (n = 80)			Indoor (n = 89)		
	Raw Data	BW 1 Hz	Manufacturer	Raw Data	BW 1 Hz	Manufacturer
25.1 m sprinting with CODs (1) (%)	1.4 ± 0.2	1.5 ± 0.2	1.8 ± 0.2	1.9 ± 0.2	1.9 ± 0.2	1.9 ± 0.2
10 m walking with CODs (2) (%)	1.7 ± 0.2	2.1 ± 0.3	1.6 ± 0.2	2.4 ± 0.3	2.5 ± 0.3	2.0 ± 0.3
10 m jogging with jump (3) (%)	1.5 ± 0.2	1.6 ± 0.2	1.3 ± 0.2	2.3 ± 0.3	2.9 ± 0.4	2.2 ± 0.3
10 m jogging (4) (%)	2.0 ± 0.3	5.4 ± 0.7	0.9 ± 0.1	3.1 ± 0.4	3.3 ± 0.4	2.8 ± 0.4
10 m walking (5) (%)	2.6 ± 0.4	6.4 ± 0.9	1.1 ± 0.1	1.6 ± 0.2	2.2 ± 0.3	1.3 ± 0.2
5 m sprinting (6) (%)	5.1 ± 0.7	4.1 ± 0.6	4.4 ± 0.6	7.1 ± 0.9	5.8 ± 0.8	6.2 ± 0.8
10 m sprinting (7) (%)	3.1 ± 0.4	2.5 ± 0.3	2.7 ± 0.4	4.3 ± 0.6	3.6 ± 0.5	3.8 ± 0.5
20 m sprinting (8) (%)	2.2 ± 0.3	2.0 ± 0.3	2.1 ± 0.3	2.3 ± 0.3	1.9 ± 0.2	2.0 ± 0.3
30 m sprinting (9) (%)	1.0 ± 0.1	0.9 ± 0.1	0.9 ± 0.1	1.5 ± 0.2	1.2 ± 0.2	1.3 ± 0.2
129.6 m entire circuit (10) (%)	1.7 ± 0.2	1.5 ± 0.2	1.1 ± 0.2	2.2 ± 0.3	2.0 ± 0.3	1.9 ± 0.2
Overall TEE (%)	2.2 ± 1.2	2.8 ± 1.9	1.8 ± 1.1	2.9 ± 1.7	2.7 ± 1.3	2.5 ± 1.5

Note: SD = Standard deviation; n = number; BW = Butterworth; COD = Change of direction. The numbers in the brackets present the section of measurement within the circuit (Figure 1).
